# Ultrarapid *BRAF* mutation detection on supernatant cell‐free DNA obtained by FNA: An accurate and expedient method for *BRAF* assessment in aggressive thyroid carcinomas

**DOI:** 10.1002/cncy.70098

**Published:** 2026-04-22

**Authors:** Jose Manuel Gutierrez Amezcua, Maria E. Arcila, Dianna L. Ng, Rusmir Feratovic, Khawaja Hasan Bilal, Jean Marc Cohen, Xiao‐Jun Wei, Brie Kezlarian‐Sachs, Carlie S. Sigel, Narasimhan Agaram, Khedoudja Nafa, Paulo Salazar, Oscar Lin, David Kim

**Affiliations:** ^1^ Department of Pathology and Laboratory Medicine Memorial Sloan Kettering Cancer Center New York New York USA; ^2^ Department of Pathology and Laboratory Medicine Larner College of Medicine at the University of Vermont and University of Vermont Medical Center Burlington Vermont USA

**Keywords:** anaplastic thyroid carcinoma, BRAF, molecular cytology, supernatant fluid

## Abstract

**Background:**

For patients with aggressive thyroid cancer, including anaplastic thyroid carcinoma (ATC) and high‐grade follicular cell‐derived non‐anaplastic thyroid carcinoma, rapid *BRAF* p.V600E testing is critical as targeted therapy with BRAF/MEK inhibitors significantly improves outcomes. This study assesses the performance and turnaround time (TAT) of the Biocartis Idylla platform for ultrarapid *BRAF* p.V600E detection across various preparations, emphasizing the use of residual CytoLyt material (supernatant cell‐free DNA [ScfDNA]).

**Methods:**

All histologically confirmed cases of aggressive thyroid carcinomas received for Idylla testing were identified. Samples were prepared either as ScfDNA, formalin‐fixed paraffin‐embedded (FFPE) cell block (CB), Diff‐Quik smear, or surgical FFPE samples. *BRAF* p.V600E testing was performed on the Idylla platform, and results were compared to a clinically validated reference method of either next‐generation sequencing (NGS) or digital‐droplet polymerase chain reaction (ddPCR). TAT for Idylla testing on ScfDNA samples was compared to FFPE preparations and to BRAF V600E immunocytochemistry (ICC).

**Results:**

Fifty‐seven samples (including 51 ATC) were tested by Idylla. The overall success rate for Idylla was 91%, with ScfDNA at 90% and surgical FFPE specimens at 100%. Of 42 samples that had NGS/ddPCR testing, concordance with the reference method showed 100% agreement (excluding failures) across all sample types. TAT for Idylla on ScfDNA samples was significantly shorter than ICC (median 2.86 vs. 46.7 hrs, *p* < .05).

**Conclusion:**

The Idylla *BRAF* assay delivers ultrarapid results that are both reliable and accurate with high success rates, particularly on ScfDNA samples. ScfDNA samples also have the fastest TAT because no histologic processing or pre‐extraction are required for testing.

## Abstract 2

Expedited *BRAF* status assessment is critical in the workup of aggressive thyroid cancer, particularly anaplastic thyroid carcinoma, to ensure timely initiation of *BRAF*‐directed therapy for eligible patients. The Idylla *BRAF* assay conducted on residual supernatant cell‐free DNA extracted from cell pellets obtained through fine‐needle aspiration delivers ultrarapid results that are both reliable and accurate with high success rate.

## INTRODUCTION

Anaplastic thyroid carcinoma (ATC) is one of the most aggressive cancers, historically carrying a uniformly dismal prognosis with median overall survival of less than 6 months.^1^ In recent years, however, the combined use of the BRAF inhibitor dabrafenib with the MEK inhibitor trametinib (DT), has completely revolutionized the management of ATC patients harboring the pathogenic *BRAF* p.V600E variant, which is present in approximately 40% of tumors.^2^ The notable increase in successful complete surgical resection following neoadjuvant use of DT and the improved overall survival relies on the timely initiation of targeted therapy, which in turn, depends on accurate and expedited *BRAF* mutation assessment.^3^, ^4^, ^5^ The consensus statement by the Facilitating Anaplastic Thyroid Cancer Specialized Treatment (FAST) group recommends that the evaluation of *BRAF* status should be done as soon as ATC is suspected and in parallel to any cytopathologic or histologic confirmation.^6^

High‐grade follicular cell‐derived non‐anaplastic thyroid carcinoma (HGFCTC) is a category of thyroid carcinomas with intermediate prognosis between ATC and well‐differentiated thyroid carcinomas of follicular origin ^7^. Similarly to ATC, a proportion of these tumors may be *BRAF* p.V600E driven. Because response to radioactive iodine is generally poor in treatment‐naïve cases, the use of DT is being explored either as part of redifferentiation therapy to induce increased radioactive iodine uptake in the tumor or to downstage surgically unresectable tumors.^8^, ^9^, ^10^ Considering the dramatic responses observed in some patients, expedited *BRAF* analysis may be critical for clinical management.^11^

Ultrasound‐guided fine‐needle aspiration (FNA) is the fastest and least invasive method for obtaining material for morphologic confirmation and ancillary testing, with the added benefit of on‐site adequacy assessment when performed by a trained cytopathologist.^12^, ^13^ *BRAF* p.V600E status is often first evaluated by immunocytochemistry (ICC) or immunohistochemistry (IHC), because it is relatively quick and inexpensive.^14^ Nevertheless, BRAF ICC testing has known pitfalls compared to molecular methods, due to interpretational challenges and variability in staining patterns (e.g., heterogeneous, weak, focal, or nonspecific background staining), and polymerase chain reaction is still deemed the standard for detecting *BRAF* variants over ICC. Per the FAST consensus statement, all BRAF immunoperoxidase results must be followed by next‐generation sequencing (NGS) molecular testing, which commonly require longer turnaround times and may have variable performance, particularly when cytologic material is limited. The reliance on cell block (CB) preparations for both ICC and molecular studies also introduces intrinsic challenges that can impact the accuracy and timeliness of results, such as longer tissue processing times, variability in ICC staining, and potentially limited material, among others.

The fully automated real‐time qualitative polymerase chain reaction (qPCR)‐based Idylla platform is designed to deliver molecular biomarker results in less than 3 hrs and has been validated to detect, among several others, *BRAF* p.V600 variants in formalin‐fixed paraffin‐embedded (FFPE) thyroid samples with high sensitivity and specificity.^15^ Although originally validated for FFPE tissue, several authors have shown that the Idylla platform performs equally well with a variety of cytology preparations,^16^, ^17^ including needle rinses from routine thyroid FNAs^18^ and residual supernatant cell‐free DNA (ScfDNA) material from liquid‐based preparations.^19^ By circumventing the need for cell block preparation, this platform offers the possibility of providing same‐day biomarker molecular results that allow the rapid implementation of targeted treatment plans, such as DT therapy initiation in eligible patients with ATC.

In this study, we evaluated the diagnostic performance and turnaround time (TAT) of Idylla *BRAF* testing on cytology samples from aggressive thyroid cancers (ATC and HGFCTC). We emphasize the use of ScfDNA, obtained from our institution’s cytopathologist‐staffed FNA clinic via an ultrarapid *BRAF* protocol, which circumvents the need for BRAF V600E ICC and preserves valuable cell block material for diagnostic assessment. The performance of Idylla *BRAF* testing on ScfDNA was assessed by comparing the results to a clinically validated reference method of either NGS by MSK‐IMPACT or digital‐droplet PCR (ddPCR). For samples that had concurrent BRAF V600E ICC performed, results and TAT were also compared to Idylla testing.

## MATERIALS AND METHODS

### Cohort selection and sample preparation

Following approval from the institutional review board (17‐634), all pathology samples of histologically confirmed ATC and HGFCTC with clinical requests for Idylla *BRAF* testing were retrospectively identified from June 2019 to December 2024 at Memorial Sloan Kettering Cancer Center. Data collected included patient demographics (age at the time of diagnosis and sex), final pathologic diagnosis, cytology preparation tested, BRAF ICC result, and TAT for ICC and Idylla *BRAF* testing. Any subsequent NGS results by the institution’s large panel matched tumor‐to‐normal assay (MSK‐IMPACT) or *BRAF* p.V600E results from ddPCR on the same tumor were recorded and used as reference to resolve discrepancies between Idylla and ICC.

All cytology samples of ATC and HGFCTC were procured by ultrasound‐guided FNA at the study institution’s cytopathologist‐staffed clinic. Samples were collected in CytoLyt fixative (Hologic, Malborough, Massachusetts) and in 10% neutral buffered formalin and sent to the cytopathology laboratory to be processed. ScfDNA samples were prepared from cell pellets obtained from residual CytoLyt material following the creation of a ThinPrep slide with or without CB creation. The corresponding ThinPrep slide was assessed as a surrogate for tumor presence and content. The remnant CytoLyt material was considered acceptable for testing if tumor cells were present at ≥10% based on visual inspection. A 50‐μL aliquot of the unextracted ScfDNA material was used for Idylla *BRAF* testing. In‐depth details about the processing of ScfDNA samples are described in a previous publication.^20^

CB preparation followed a modified method from samples either submitted in 10% neutral buffered formalin or CytoLyt that incorporates the use of 95% ethanol before the standard HistoGel (Thermo Scientific, Waltham, Massachusetts) cell block preparation.^21^ Surgical samples (core biopsies or resection specimens) were all fixed in 10% neutral buffered formalin fixative and were paraffin embedded. All FFPE specimens (CB and surgical cases) used one to five unstained sections (5‐μm thick) submitted on glass slides for Idylla testing. A corresponding hematoxylin–eosin (H & E) stained section was used to assess adequacy and tumor fraction. Manual macrodissection was performed to enrich for tumor, when possible and necessary. Samples were rejected for molecular testing if the tumor proportion was <10% and the sample was not amenable to manual enrichment.

### Ultrarapid *BRAF* p.V600 assessment by the Idylla platform

Details of the Idylla assay have been previously described.^15^ Briefly, the Idylla *BRAF* assay is a fully automated cartridge‐based real‐time qPCR assay that provides ultrarapid mutation assessment in approximately 2 hrs. Pre‐extraction of DNA is not required as the platform incorporates all the necessary steps, from deparaffinization (for FFPE samples) to cell lysis, nucleic acid extraction, amplification, and mutation detection in a single cartridge. The cartridge may be loaded with a wide range of tumor sources and preparations, including fresh cellular suspensions, concentrated supernatants and FFPE tissue scrapings. A tumor purity estimate of >10% by manual review of either the corresponding H & E for FFPE unstained tissue slides or corresponding ThinPrep for ScfDNA samples is required for testing. Tumor purity is the estimated proportion of tumor cells within a sample, calculated as the percentage of tumor nuclei relative to all nuclei present. Samples with a lower tumor purity are rejected. Once the process is completed, the PCR amplification curves are visualized along with the quality control standards of total (wild‐type internal control) and mutant *BRAF* cycles of quantification (Cq), as well as the difference between these two signals (ΔCq). A minimum *BRAF* wild‐type internal control Cq value of 36 (∼25 ng of DNA) is required for successful Idylla testing for a limit of detection of 2.5% variant allele frequency. Samples with a Cq >36 are considered invalid and reported as a test failure due to low DNA content. The results are reviewed by a board‐certified practicing molecular pathologist before being released.

### Comparison of accuracy and turn‐around time between Idylla and ICC

To evaluate the performance of the Idylla *BRAF* assay on ScfDNA samples, results were compared against three clinically validated reference methods (ICC, NGS, and ddPCR) performed on the same tumor specimen used for Idylla testing. BRAF V600E immunoperoxidase stain was performed using a BRAF p.V600E mutation–specific antibody (mouse monoclonal, clone VE1; dilution 1:400, Spring Bioscience, Pleasanton, California) on the Leica Bond III automatic platform (Leica Biosystems, Wetzlar, Germany). The BRAF V600E immunoperoxidase stains were interpreted and reported by a head and neck pathologist for surgical samples or a cytopathologist for cytology samples. The results were interpreted as follows: positive (defined as strong, diffuse cytoplasmic staining of tumor cells), negative (no cytoplasmic staining in tumor cells), or equivocal (weak and/or focal cytoplasmic staining). Equivocal and noncontributory ICC results were designated as discordant to the expected result.

NGS and ddPCR results were used as the reference standard for determination of *BRAF* mutation status in assessing Idylla assay performance across specimen types. NGS testing was performed using MSK‐IMPACT, a hybridization capture‐based, matched tumor–normal targeted sequencing assay interrogating all coding exons and selected introns of 505 cancer‐associated genes. Cases were required to demonstrate a minimum tumor purity of >10% by manual histopathologic review and a minimum DNA yield of 0.9 ng/mL post extraction. Detailed descriptions of the MSK‐IMPACT assay and its validated performance on cytology specimens have been previously reported.^22^, ^23^

Samples were reflexively analyzed by ddPCR when a *BRAF* p.V600E variant was suspected at levels below threshold on NGS. ddPCR was performed using extracted DNA partitioned into droplets with an Automated Droplet Generator (Bio‐Rad Laboratories, Hercules, California). PCR amplification was performed on a C1000 Touch thermal cycler (Bio‐Rad Technologies). A minimum DNA input of 10 ng was required to achieve an analytical sensitivity of 0.1%. Wild‐type and mutant *BRAF* p.V600 probes were labeled with HEX and FAM fluorophores, respectively (#10049550; Bio‐Rad Laboratories). A positive result was defined by the presence of at least five droplets forming a discrete cluster within the *BRAF* p.V600E mutant quadrant.

TAT was defined as the sum of sample preparation and test run time for *BRAF* analysis. For ICC/IHC, TAT included the time required to process either a FFPE tissue block or CB, in addition to immunoperoxidase staining and processing period. Specifically, ICC processing time was measured from the moment the stain was ordered by the pathologist (after confirming adequate material in CB), until slide verification and release by the laboratory. For Idylla, TAT was calculated from specimen receipt in the molecular laboratory to the availability of results for review by a molecular pathologist. When ScfDNA was used for Idylla *BRAF* testing, the time required for ScfDNA sample preparation was added to the Idylla run time.

### Statistical analysis

Statistical analysis for group comparisons of continuous data was performed using a two‐tailed Mann‐Whitney test. A Pearson’s χ^2^ test was performed for comparing three or more categorical groups. A Fisher’s exact test was performed for comparing two categorical groups. All boxplots show the median (center line with value), 25th and 75th percentiles (bounding box) along with the 1.5 interquartile range (“whiskers”). Statistical significance was set at *p* < .05. All statistics and graphical representations were performed using R project.

## RESULTS

### Patient demographics and specimen characteristics

**FIGURE 1:**
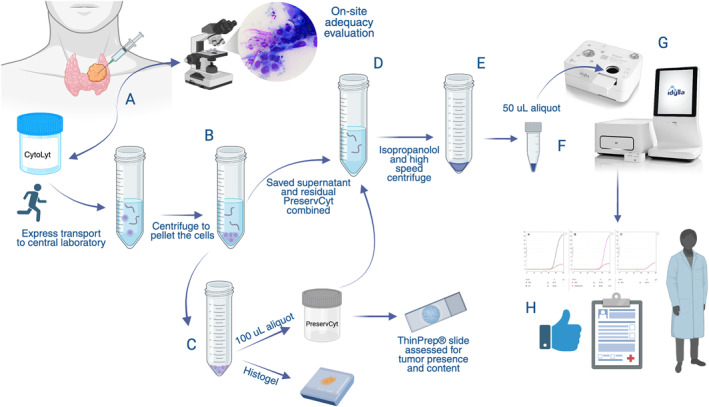
Ultrarapid BRAF protocol at Memorial Sloan Kettering Cancer Center. Key steps in the process are illustrated. (A) The specimen is collected at our cytopathologist‐staffed fine‐needle aspiration clinic and after adequacy assessment it is transported by express courier for expedited accessioning and processing. (B) CytoLyt contents are transferred to a conical tube and pelleted. (C) The cell pellet is resuspended and used for cell block creation and ThinPrep slide processing. (D) The CytoLyt supernatant and residual PreservCyt are combined. (E) Isopropanol is added to precipitate cell‐free DNA, followed by centrifugation. (F) The pelleted material is then sent to the molecular laboratory for expedited accessioning and processing. (G) The pellet is resuspended and a 50‐uL aliquot is placed into the Idylla *BRAF* mutation assay cartridge and processed as per the manufacturer’s protocol. (H) The Idylla results are verified by a board‐certified practicing molecular pathologist before being released to the patient and clinicians. Created in BioRender (https://BioRender.com/w5gphag).

We analyzed 57 samples from 56 patients, comprising 51 ATC (89%) and six HGFCTC (11%). Patient demographics are summarized in Table 1. Sample types included 30 ScfDNA (53%) that underwent our ultrarapid *BRAF* protocol (Figure 1), one Diff‐Quik stained smear slide (2%), 16 CB (28%), and 10 FFPE surgical biopsies/resection specimens (17%). The majority of the cytology samples (*n* = 33, 70%) were classified as category VI (malignant) according to The Bethesda System for Reporting Thyroid Cytopathology (TBSRTC), 13 samples (28%) were classified as malignant without a specific TBSRTC category, and one sample (2%) was classified as TBSRTC category IV. A detailed explanation of final diagnoses, diagnostic categories, and immunoperoxidase workup performed on all samples is available in Table S1.

**TABLE 1:** Cohort demographics by material tested.

Characteristic	Overall, *N* = 57	ScfDNA, *N* = 30	Diff‐Quik, *N* = 1	Cell block, *N* = 16	Surgical sample, *N* = 10	*p* ^a^
Age, years, median (IQR)	68 (58–76)	70 (60–80)	68 (68–68)	66 (57–74)	69 (59–73)	.8
Sex, No. (%)						.14
Female	27 (47)	17 (57)	0 (0)	8 (50)	2 (20)	
Male	30 (53)	13 (43)	1 (100)	8 (50)	8 (80)	
Idylla result, No. (%)						.7
Failed	5 (9)	3 (10)	0 (0)	2 (12)	0 (0)	
Successful	52 (91)	27 (90)	1 (100)	14 (88)	10 (100)	
Tumor type, No. (%)						.5
ATC	51 (89)	26 (87)	1 (100)	14 (88)	10 (100)	
HGFCTC	6 (11)	4 (13)	0 (0)	2 (12)	0 (0)	

Abbreviations: ATC, anaplastic thyroid carcinoma; HGFCTC, high‐grade follicular cell‐derived non‐anaplastic thyroid carcinoma; ScfDNA: supernatant cell‐free DNA.

^a^ Kruskal–Wallis rank sum test; Fisher exact test.

Corresponding *BRAF* testing was available for all samples by at least one additional modality of either BRAF V600E immunoperoxidase (ICC/IHC), NGS, or ddPCR (Table 2). Of these, 95% (*n* = 54) were performed on the same tissue tested by Idylla. Three samples had orthogonal testing on a subsequent excision sample because limited material was available at the time of Idylla testing. For performance assessment, 42 (74%) samples underwent testing by both Idylla and a molecular reference standard (NGS or ddPCR), of which 15 harbored *BRAF* mutations. Among these, 32 were also tested by ICC/IHC, enabling direct three‐way comparison.

**TABLE 2:** Concordance of *BRAF* V600E immunoperoxidase (ICC/IHC), Idylla results for all sample types, and Idylla results for residual CytoLyt supernatant samples (ScfDNA), compared to NGS results.

	NGS results		
	Positive	Negative	Not available
*BRAF* ICC/IHC results			
Positive	10	1	5
Negative	0	16	8
Equivocal	1	2	0
Idylla *BRAF* results (overall)			
Positive	13	1	6
Negative	0	25	2
Failure	1	2	2
Idylla *BRAF* results (ScfDNA)			
Positive	7	0	2
Negative	0	14	4
Failure	0	2	1

Abbreviations: ICC, immunocytochemistry; IHC, immunohistochemistry; NGS, next‐generation sequencing; ScfDNA, supernatant cell‐free DNA.

### Analytical performance for Idylla *BRAF* testing

**FIGURE 2:**
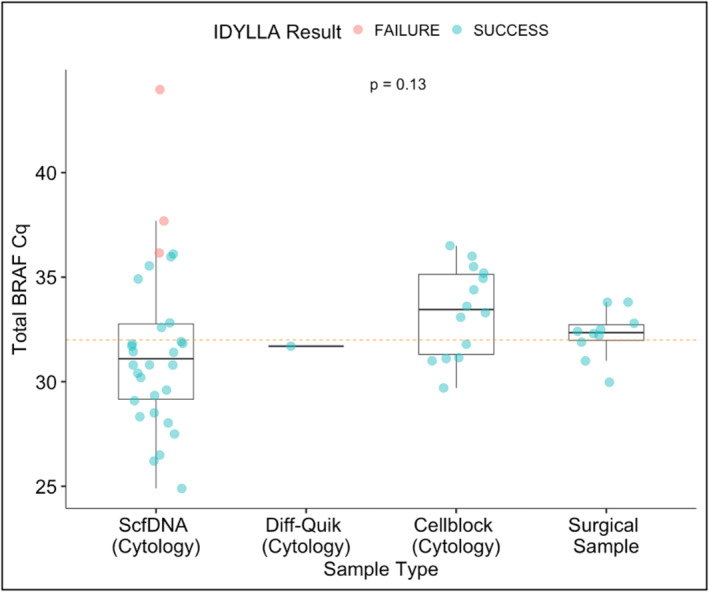
Total *BRAF* Cq results tested by Idylla by sample preparation. Samples that failed testing due to low amplification are noted by a red circle whereas those that were successful are blue. Boxplots show the median (center line with value), 25th and 75th percentiles (bounding box) along with the 1.5 interquartile range. The dashed horizontal line indicates a total *BRAF* Cq of 33, samples above this line indicate low amplification with concerns for false‐negative results.

Of the total cohort, 52 (91%) samples were successfully tested by Idylla; five failed or were not performed due to low tumor content (either low tumor proportion or scant cellularity). The median total *BRAF* Cq for all preparations was 31.82 (range, 24.9–43.97). When stratified by preparation type, median total *BRAF* Cq was 31.1 (range, 24.9–43.97) for ScfDNA, 33.5 (range, 29.7–36.5) for CB, and 32.35 (range, 29.97–33.8) for surgical FFPE material (*p* = .24) (Figure 2). Among the failures, two CB samples were failed before testing due to insufficient tumor cells on manual review whereas three ScfDNA samples failed testing due to low amplification secondary to low nucleic acid quality/quantity. Cytology samples (ScfDNA, Diff‐Quik, and CB) showed an overall 89% (42 of 47) test success rate.

Success rates by sample preparation were 90% for ScfDNA (27 of 30), 100% for Diff‐Quik (one of one), 88% for CB (14 of 16), and 100% for surgical FFPE material (10 of 10). The Diff‐Quik sample performed successfully with a Cq value of 31.7 with no *BRAF* variant detected. Because of the limited cellularity of this sample, additional testing could not be performed aside from Idylla. Nevertheless, confirmatory testing by NGS was performed on a subsequent surgical resection specimen and found no *BRAF* variant, in concordance with the initial Idylla results. Success rates in relation to tumor cellularity showed that successful Idylla samples had a median tumor percentage of 40% (range, 10%–90%) whereas samples that failed testing had a median tumor percentage of 20% (range, 10%–60%) (*p* = .11). *BRAF* variant detection rates were 33% (9 of 27) for ScfDNA, 50% (7 of 14) for cytology CB, and 40% (4 of 10) for surgical FFPE samples with an overall total detection rate of 39%.

### Concordance analysis of *BRAF* p.V600E detection across Idylla, ICC/IHC, NGS, and ddPCR

NGS was ordered and performed on 74% (42 of 57) of the total cases, which included 23 ScfDNA, 12 cytology CB, one Diff‐Quik smear, and six surgical specimens. Among the NGS‐tested samples, 36% (15 of 42) were positive for a *BRAF* p.V600E variant, whereas 64% (27 of 42) were wild‐type. *BRAF* ddPCR was performed on one CB (sample 39) that showed a *BRAF* mutation below the threshold for NGS calling, but was confirmed to be present by ddPCR, in concordance with the initial Idylla result. Three of 42 (7%) Idylla samples with follow‐up NGS failed due to low amplification. The overall concordance of Idylla *BRAF* with NGS/ddPCR was 100% for the 39 (93%) successfully tested Idylla samples.

**FIGURE 3:**
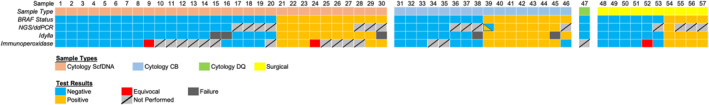
Oncoplot of the samples tested by Idylla *BRAF* showing: sample type, *BRAF* status determined by NGS/ddPCR (reference method), and results of Idylla, BRAF V600E immunoperoxidase, and NGS/ddPCR testing. ddPCR indicates digital‐droplet polymerase chain reaction; NGS, next‐generation sequencing.

Thirty of the 42 (71%) Idylla samples with follow‐up NGS also had BRAF immunoperoxidase reported for comparison. Of these, three samples had the ICC/IHC reported as equivocal/noncontributory. For these three equivocal cases, Idylla *BRAF* testing was successfully performed and showed full concordance with NGS results. The remaining ICC/IHC results were 100% concordant with the NGS findings (Figure 3).

### Turnaround time comparison between Idylla BRAF and ICC/IHC

**FIGURE 4:**
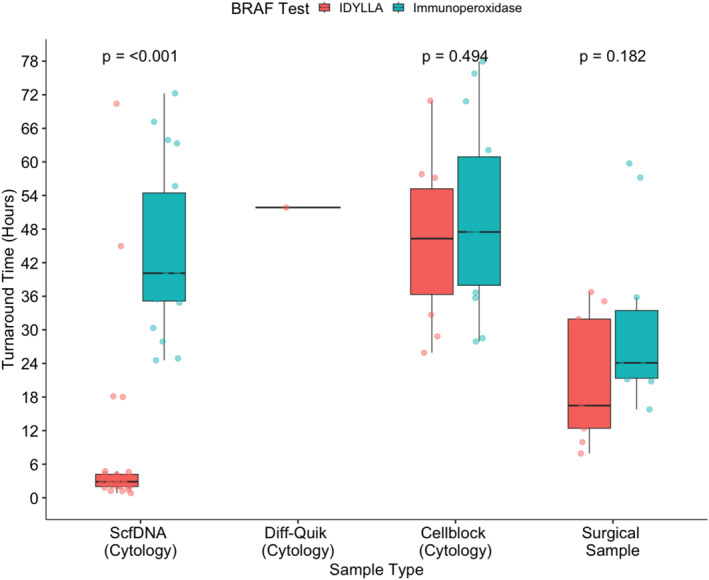
Turnaround time comparison between Idylla and immunoperoxidase (IHC/ICC) by sample preparation. Supernatant cell‐free DNA samples had concurrent ICC performed on a cell block prepared from the same specimen tested by Idylla. Cytology cell block and surgical samples had unstained slides cut from the same FFPE block for both Idylla and ICC/IHC testing. Boxplots show the median (center line with value), 25th, and 75th percentiles (bounding box) along with the 1.5 interquartile range. FFPE indicates formalin‐fixed paraffin‐embedded; ICC, immunocytochemistry; IHC, immunohistochemistry.

The median TAT for ScfDNA samples that underwent the ultrarapid *BRAF* protocol was 2.86 hrs (range, 0.82–70.4 hrs) compared to 46.7 hrs (range, 24.5–102 hrs) for BRAF ICC on their corresponding CB preparation (*p* < .05). For CB samples, the median TAT on Idylla was 50.4 hrs (range, 25.9–93.8 hrs) and BRAF ICC was 47.5 hrs (range, 27.9–77.9 hrs) (*p* > .05). Similarly, for surgical FFPE samples the median TAT for Idylla was 20.4 hrs (range, 7.91–128 hrs) and BRAF IHC was 24.1 hrs (range, 15.8–59.7 hrs) (*p* > .05). A direct comparison could not be performed for the Diff‐Quik smear, which showed an Idylla TAT of 50.7 hrs, because there was insufficient material for additional testing (Figure 4).

## DISCUSSION

In patients with ATC, timely initiation of targeted therapy has a direct impact on patient management and overall survival.^3^, ^4^, ^5^ FNA currently represents one of the fastest and least invasive methods for tissue sampling for ATC. BRAF V600E ICC, which offers high sensitivity and specificity, is often the preferred initial method for assessing *BRAF* status.^14^ However, BRAF ICC has several limitations over molecular testing. ICC performed on direct smears has been noted to have high false‐positive rates,^24^ whereas ICC on CB introduces additional processing time and is still subject to inaccurate results because of high background staining, or focal, weak cytoplasmic staining.^25^, ^26^ Extensive necrosis or inflammation, frequently seen in ATC samples, may also preclude ICC interpretation.^27^ To circumvent these limitations, this study evaluates the performance of Idylla *BRAF* testing in aggressive thyroid carcinomas, with a cohort particularly enriched for ATC.

Overall, this study demonstrated that Idylla *BRAF* testing had a higher concordance and significantly faster TAT than BRAF ICC when compared to ddPCR and/or NGS. Four cases demonstrated discordance between BRAF V600E ICC/IHC and molecular Idylla *BRAF* testing. In two cases, ICC was interpreted as equivocal or negative whereas Idylla testing was positive (samples 24 and 46, respectively), and in two cases ICC/IHC were equivocal or noncontributory whereas Idylla yielded a definitive negative result (samples 9 and 52). In all such cases, Idylla results were corroborated by NGS performed on the same or a subsequent specimen, supporting the high analytic accuracy of the assay. Discordances were not enriched in any single specimen type and were observed across cell‐free DNA (*n* = 2), cell block (*n* = 1), and surgical core biopsy (*n* = 1).

Although BRAF V600E immunoperoxidase stain demonstrates high reported sensitivity and specificity in thyroid carcinoma, including cytology specimens, definitive positive interpretation requires strong and diffuse cytoplasmic tumor cell staining.^14^, ^25^ Weak or focal staining patterns as seen in the discordant samples may lead to equivocal results and reduced reliability, underscoring the importance of molecular confirmation in such cases to minimize false‐negative interpretation.^28^ Besides demonstrating that Idylla testing on ScfDNA is the fastest method for assessing *BRAF* status with a high success rate, there are additional noteworthy advantages to this testing method. First, methanol‐based fixation produces better DNA quality and yield for molecular studies, because it bypasses the deleterious effects on DNA structure and integrity induced by formalin fixation.^29^ Additionally, because ScfDNA samples are processed individually in a closed system, they are less susceptible to cross‐contamination associated with batching and pooling of multiple samples, a potential risk in CB or FFPE samples.^23^

Pairing the more streamlined processing and higher quality DNA yield from ScfDNA samples with the Idylla platform ensures a quicker result. The Idylla platform is a simple to use, single cartridge qPCR test for detecting specific molecular variants. Because no separate DNA extraction step is required, manual hands‐on‐time is approximately 2–5 minutes.^16^, ^30^, ^31^ The time to run the assay, from placing the single use cartridge to result, takes approximately 2–3 hrs. The short hands‐on‐time and run time allows for the ability to assess for *BRAF* p.V600 variants under 24 hrs from biopsy. Therefore, any hospital or reference laboratory can implement the Idylla platform without the need for a fully staffed molecular laboratory. In comparison, targeted NGS panels can take weeks to result, a waiting time that some patients with aggressive thyroid carcinomas, particularly ATC, cannot afford. An important limitation to the Idylla platform is that only a small panel of mutations at specific loci can be evaluated based on cartridge design. However, in the context of aggressive thyroid cancers where a single mutation is of critical importance to initiate targeted therapy, the Idylla platform is well suited for this task. Although p.V600E is the predominant *BRAF* alteration in thyroid carcinoma, less common non‐V600E variants such as p.K601E, p.V600K, and p.K601G have been described.^32^, ^33^, ^34^ Although the Idylla *BRAF* assay does not differentiate among substitutions of the same codon (e.g., p.V600E/D and p.V600K/R/M), in our cohort, all Idylla‐positive cases with available NGS follow‐up were confirmed to harbor p.V600E, and no p.V600D variants were identified. Notably, p.V600D has not been reported in thyroid carcinoma to date.^35^, ^36^ Therefore, in the context of thyroid malignancies, a positive Idylla *BRAF* result detecting p.V600E/D is highly likely to represent a p.V600E mutation.

The results from our experience using FNA‐derived ScfDNA samples of aggressive thyroid carcinomas on the Idylla platform demonstrate that it is a reliable method for molecularly assessing *BRAF* variants with the quickest TAT. However, there are a few limitations to this study. Namely, not all samples had all three methods of *BRAF* testing performed (i.e., Idylla, ICC, and NGS) for comparison. Additionally, some samples had *BRAF* assessment by immunoperoxidase and/or NGS on a separate sample of the same tumor which may have a different tumor yield affecting testing. Last, although this is the first cohort to date analyzing Idylla *BRAF* performance on aggressive thyroid carcinomas, the overall number of samples is still low. Future studies with larger cohorts of *BRAF* testing via a variety of methods on the same material would help support the findings of this study.

In conclusion, the Idylla *BRAF* assay, conducted on residual ScfDNA extracted from cell pellets via FNA, is both a reliable and accurate method of *BRAF* status evaluation. In our experience, it is also the fastest available option for any cytology sample preparation compared to ICC. This ultrarapid method for determining *BRAF* status addresses current critical gaps in molecular testing to ensure timely initiation of *BRAF*‐directed therapy for eligible patients undergoing workup for aggressive thyroid malignancies, particularly anaplastic thyroid carcinoma.

## AUTHOR CONTRIBUTIONS

**Jose Manuel Gutierrez Amezcua**: Conceptualization; investigation; methodology; writing—original draft; writing—review and editing; formal analysis; visualization; resources; data curation. **Maria E. Arcila**: Project administration; validation; formal analysis; writing—review and editing; visualization. **Dianna L. Ng**: Writing—review and editing; funding acquisition; resources; visualization. **Rusmir Feratovic**: Project administration; validation. **Khawaja Hasan Bilal**: Data curation; resources. **Jean Marc Cohen**: Supervision and resources. **Xiao‐Jun Wei**: Supervision and resources. **Brie Kezlarian‐Sachs**: Writing—review and editing; supervision; resources. **Carlie S. Sigel**: Supervision; resources. **Narasimhan Agaram**: Supervision and resources. **Khedoudja Nafa**: Supervision; validation; project administration. **Paulo Salazar**: Validation and project administration. **Oscar Lin**: Supervision. **David Kim**: Conceptualization; investigation; validation; formal analysis; data curation; writing—original draft; writing—review and editing; resources; supervision; project administration; software; visualization; methodology; and funding acquisition.

## CONFLICT OF INTEREST STATEMENT

Maria E. Arcila reports advisory or consulting fees from Axis Medical Education, Clinical Education Alliance, LLC, Merck Sharp & Dohme, PeerView Institute for Medical Education (PVI), Physicians’ Education Resource, RMEI Medical Education, LLC, AstraZeneca, Biocartis, i3 Health, and Roche; fees for professional services from AstraZeneca; and intellectual property rights in SOPHiA GENETICS S.A. Oscar Lin reports consulting fees from Hologic and Janssen Biotech. The other authors declare no conflicts of interest.

## Supporting information

Supporting Information S1
