# Supplementary material for: Ultrarapid BRAF mutation detection on supernatant cell‐free DNA obtained by FNA: An accurate and expedient method for BRAF assessment in aggressive thyroid carcinomas
Source: Cancer Cytopathol. 2026 Apr 22;134(5):e70098. doi: 10.1002/cncy.70098 (PMC13102139; doi:10.1002/cncy.70098)
Supplement: Supplementary file 1 — Supporting Information S1 [file CNCY-134-0-s001.docx]

| Sample | Sample tested with Idylla^TM^ | TBSRTC category | Diagnosis | Immunoperoxidase stains  Positive Negative Equivocal/NC | | |
| --- | --- | --- | --- | --- | --- | --- |
| 1 | ScfDNA | N/A | High-grade carcinoma | PanCK, p40 | BRAF V600E, TTF-1, TG, SYN, CAL | PAX-8 |
| 2 | ScfDNA | 6 | Compatible with ATC | PanCK, TG (f), TTF-1 (f), PAX-8 (f) | BRAF V600E, CAL |  |
| 3 | ScfDNA | 6 | Carcinoma of thyroid follicular origin. Cannot rule out a more aggressive lesion^e^ | TTF-1 | BRAF V600E | TG, PAX-8 |
| 4 | ScfDNA | N/A | Consistent with ATC | PAX-8 (f) | BRAF V600E, TTF-1, TG, NRAS Q61R |  |
| 5 | ScfDNA | 6 | Undifferentiated malignant neoplasm, could represent ATC with osteoclast-like giant cells | NRAS Q61R | PanCK, Cam 5.2, CK7, TG, PAX-8, TTF-1, CAL, SYN, p40, Napsin-A, BRAF V600E |  |
| 6 | ScfDNA | 6 | HGFCTC, follicular carcinoma phenotype in this material | PAX-8, TTF-1, TG (f) | BRAF V600E, NRAS Q61R |  |
| 7 | ScfDNA | 6 | Most consistent with poorly differentiated oncocytic (Hurthle cell) carcinoma | PAX-8, TTF-1, PanCK, TG (r) | BRAF V600E, CD20, CD3 |  |
| 8 | ScfDNA | 6 | High-grade carcinoma, could represent ATC | OSCAR | BRAF V600E, PAX-8, TTF-1, CAL, TG, CK7, CK19, CD45, PAX-5, SOX10, S100, Melan-A, HMB45 |  |
| 9 | ScfDNA | 6 | Papillary carcinoma with transformation to anaplastic thyroid carcinoma | PAX-8 | TTF-1, TG (in ATC component)^a^ | BRAF V600E |
| 10 | ScfDNA | 6 | Scant malignant epithelioid and spindle cells present^b^ |  |  |  |
| 11 | ScfDNA | 6 | Consistent with ATC | PAX-8 | TTF-1, TG |  |
| 12 | ScfDNA | 6 | Compatible with ATC | PanCK | TTF1, PAX-8, TG |  |
| 13 | ScfDNA | 6 | Consistent with ATC | PanCK | TTF-1, PAX-8, TG |  |
| 14 | ScfDNA | N/A | Carcinoma with high-grade features and squamous differentiation | PAX-8, p40 (f) | TTF-1, TG, WT-1 |  |
| 15 | ScfDNA | N/A | Carcinoma of thyroid origin | TTF-1, PAX-8, TG, NRAS Q61R |  |  |
| 16 | ScfDNA | 6 | Malignant spindle and epithelioid neoplasm, compatible with ATC |  | PAX-8, TTF-1, TG, BRAF V600E, PanCK, CK18, SOX10, Desmin, ERG |  |
| 17 | ScfDNA | 4 | Hurtle cell carcinoma^c^ | PanCK, TTF-1, PAX-8, TG | BRAF V600E |  |
| 18 | ScfDNA | N/A | Poorly differentiated carcinoma with anaplastic features. | PanCK, Cam 5.2 (f) | TTF-1, PAX-8, TG, BRAF V600E, SOX10 |  |
| 19 | ScfDNA | 6 | ATC | PAX-8 | TG, TTF-1, BRAF V600E |  |
| 20 | ScfDNA | 6 | Papillary thyroid carcinoma with oncocytic features^d^ | PAX-8, TTF-1 | ER, GATA-3, CAL, SYN, CHR |  |
| 21 | ScfDNA | 6 | Consistent with ATC | PAX-8, BRAF V600E | TTF-1, TG |  |
| 22 | ScfDNA | 6 | Malignant thyroid neoplasm, biphasic. Transformation into ATC should be considered | TG, TTF-1, PAX8-, BRAF V600E |  |  |
| 23 | ScfDNA | 6 | Consistent with ATC | PanCK, PAX-8 (f), BRAF V600E (f) | TTF-1, TG |  |
| 24 | ScfDNA | 6 | Most consistent with ATC | PAX-8, PanCK(f), Cam 5.2 (f) | TTF-1, TG, CAL | BRAF V600E |
| 25 | ScfDNA | 6 | Consistent with ATC | PAX-8 | TTF-1, TG |  |
| 26 | ScfDNA | N/A | Consistent with ATC | PAX-8 (f) | TTF-1, TG |  |
| 27 | ScfDNA | N/A | Carcinoma with anaplastic features^b^ |  |  |  |
| 28 | ScfDNA | 6 | Malignant spindle and giant cell neoplasm, compatible with ATC | SATB2 | PAX-8, TTF-1, PanCK, CAL, SYN | TG |
| 29 | ScfDNA | 6 | Compatible with ATC | PanCK, TTF-1, PAX-8, BRAF V600E | p40, TG |  |
| 30 | ScfDNA | 6 | Favor ATC | PAX-8, TTF-1 (f), BRAF V600E | TG |  |
| 31 | CB | 6 | Compatible with ATC | PanCK, PAX-8 | TG, TTF-1, BRAF V600E |  |
| 32 | CB | N/A | Poorly differentiated carcinoma | TTF-1, PAX-8 | TG, Napsin-A, BRAF V600E |  |
| 33 | CB | N/A | High-grade carcinoma of thyroid origin | TTF-1 (f), CK7, PAX-8 | TG, BRAF V600E |  |
| 34 | CB | 6 | High-grade carcinoma consistent with ATC | PAX-8 | TTF-1, TG |  |
| 35 | CB | 6 | Differential diagnosis includes (and is not limited to) poorly differentiated thyroid carcinoma | CK7, TTF-1, PAX-8, TG | ER, GATA-3 |  |
| 36 | CB | 6 | High-grade carcinoma, compatible with ATC | PanCK, Cam5.2 (f), PAX-8 (f), TTF-1 (r) | BRAF V600E |  |
| 37 | CB | 6 | Hurtle cell carcinoma^e^ | PanCK, OSCAR, TTF-1, PAX-8, TG | Cam 5.2, SYN, CAL, BRAF V600E |  |
| 38 | CB | N/A | Poorly differentiated carcinoma with anaplastic features | PanCK | TTF-1, PAX-8, TG, BRAF V600E |  |
| 39 | CB | 6 | ATC | PAX-8, TTF-1 (f), TG (r), BRAF V600E |  |  |
| 40 | CB | 6 | Undifferentiated malignant neoplasm; compatible with ATC | BRAF V600E, TTF1 (r), PAX-8 (r) | CAM5.2, PanCK, TG, SOX10 |  |
| 41 | CB | 6 | ATC | TTF-1 (r), PAX-8 (f), BRAF V600E | TG |  |
| 42 | CB | 6 | Consistent with ATC with squamous differentiation | CK5/6, p40, BRAF V600E (f), PAX-8 (f) | TTF-1, TG |  |
| 43 | CB | N/A | Most consistent with ATC | PanCK, p40, PAX-8 (f), BRAF V600E (f) | TTF-1, TG |  |
| 44 | CB | 6 | Carcinoma with squamous features, consistent with ATC with an associated better differentiated component | BRAF V600E, PanCK, CK7, PAX-8, p40 | TTF-1, TG (in ATC component)^a^ |  |
| 45 | CB | 6 | Carcinoma morphologically suggestive of ATC | BRAF V600E (f) | TTF-1, PAX-8 |  |
| 46 | CB | N/A | Carcinoma. raises possibility of ATC | PAX-8 (f) | TTF-1, GATA-3, ER, PR, BRAF V600E |  |
| 47 | Diff-Quik | N/A | Carcinoma compatible with ATC arising from PTC (historical) | N/A | N/A | N/A |
| 48 | Surgical | N/A | ATC with better differentiated Hurtle cell carcinoma | PanCK | TTF-1, TG, PAX-8 (in ATC component)^a^ BRAF V600E |  |
| 49 | Surgical | N/A | Undifferentiated (anaplastic) carcinoma without residual well-differentiated component | PanCK, CK18, Cam 5.2, 34BE12 | TTF-1, PAX8, TG, S100, HMB45, BRAF V600E |  |
| 50 | Surgical | N/A | Undifferentiated malignant neoplasm consistent with ATC |  | PanCK, PAX-8, TTF-1, TG, CK19, CK5/6, BRAF V600E, NRAS Q61R |  |
| 51 | Surgical | N/A | Carcinoma with spindle cell features most consistent with ATC | PAX-8, PanCK, CK18 | TG, TTF-1, Cam 5.2, S100, HMB45, Melan-A, BRAF V600E |  |
| 52 | Surgical | N/A | Metastatic high-grade carcinoma, morphologically similar to prior^b^ | PanCK (r), TTF-1, PAX-8, CK7 (r), CAM5.2 (r) | Napsin-A, NRAS Q61R | BRAF V600E |
| 53 | Surgical | N/A | Compatible with ATC |  | PanCK, TTF-1, PAX-8, TG, BRAF V600E |  |
| 54 | Surgical | N/A | ATC | PAX-8, BRAF V600E |  |  |
| 55 | Surgical | N/A | Consistent with ATC | BRAF V600E | CD117, CK7, NRAS Q61R, PanCK, p40, TG, TTF-1, PAX-8, Cam 5.2, Melan-A, HMB-45 | S100 |
| 56 | Surgical | N/A | ATC | PAX-8, PanCK, p40, BRAF V600E | TTF-1, TG |  |
| 57 | Surgical | N/A | Undifferentiated (anaplastic) carcinoma with residual well-differentiated component of papillary tall cell phenotype | PAX-8, BRAF V600E | TTF-1, TG, (in ATC component)^a^ |  |

Supplemental Table 1: All specimens tested by Idylla^TM^ with concurrent diagnostic categories/diagnoses and immunoperoxidase work-up performed. *A*, Non-anaplastic (well or poorly differentiated) components in these samples showed immunoreactivity for TTF-1, TG, and PAX-8. *B*, Morphologically similar to prior material diagnosed as ATC. *C*, Subsequent surgical resection showed poorly differentiated component. *D*, Subsequent surgical resection showed HGFCTC with papillary nuclear phenotype. *E*, Subsequent surgical resection showed ATC.

Abbreviations: ATC: Anaplastic Thyroid Carcinoma, BRAF: v-Raf murine sarcoma viral oncogene homolog B, CAL: Calcitonin, CB: Cellblock, CD: Cluster of Differentiation, CHR: Chromogranin, CK: Cytokeratin, ER: Estrogen receptor, (f): Focal, HGFCTC: High-Grade Follicular Cell-Derived Non-Anaplastic Thyroid Carcinoma, HMB: Human Melanoma Black, N/A: NC: Non-contributory, Not applicable, NRAS: Neuroblastoma Rat sarcoma virus, PanCK: Pancytokeratin, PAX: Paired box gene, PTC: Papillary thyroid carcinoma, PR: Progesterone receptor, (r): Rare cells, ScfDNA: Supernatant cell free DNA, SYN: Synaptophysin, SOX: SRY-related HMG-box gene, TBSRTC: The Bethesda System for Reporting Thyroid Cytopathology, TG: Thyroglobulin, TTF-1: Thyroid Transcription Factor-1, WT-1: Wilms' Tumor 1.
